# A culture-, amplification-independent, and rapid method for identification of pathogens and antibiotic resistance profile in bovine mastitis milk

**DOI:** 10.3389/fmicb.2022.1104701

**Published:** 2023-01-06

**Authors:** Asal Ahmadi, Abdolrahman Khezri, Håvard Nørstebø, Rafi Ahmad

**Affiliations:** ^1^Department of Biotechnology, Inland Norway University of Applied Sciences, Hamar, Norway; ^2^TINE SA, Oslo, Norway; ^3^Institute of Clinical Medicine, Faculty of Health Sciences, UiT–The Arctic University of Norway, Tromsø, Norway

**Keywords:** mastitis, staphylococcus aureus, udder infections, nanopore sequencing technology, culture-independent sequencing, rapid diagnosis, antibiotic resistance, milk

## Abstract

**Introduction:**

Rapid and accurate diagnosis of causative pathogens in mastitis would minimize the imprudent use of antibiotics and, therefore, reduce the spread of antimicrobial resistance. Whole genome sequencing offers a unique opportunity to study the microbial community and antimicrobial resistance (AMR) in mastitis. However, the complexity of milk samples and the presence of a high amount of host DNA in milk from infected udders often make this very challenging.

**Methods:**

Here, we tested 24 bovine milk samples (18 mastitis and six non-mastitis) using four different commercial kits (Qiagens’ DNeasy^®^ PowerFood^®^ Microbial, Norgens’ Milk Bacterial DNA Isolation, and Molzyms’ MolYsis™ Plus and Complete5) in combination with filtration, low-speed centrifugation, nuclease, and 10% bile extract of male bovine (Ox bile). Isolated DNA was quantified, checked for the presence/absence of host and pathogen using PCR and sequenced using MinION nanopore sequencing. Bioinformatics analysis was performed for taxonomic classification and antimicrobial resistance gene detection.

**Results:**

The results showed that kits designed explicitly for bacterial DNA isolation from food and dairy matrices could not deplete/minimize host DNA. Following using MolYsis™ Complete 5 + 10% Ox bile + micrococcal nuclease combination, on average, 17% and 66.5% of reads were classified as bovine and *Staphylococcus aureus* reads, respectively. This combination also effectively enriched other mastitis pathogens, including *Escherichia coli* and *Streptococcus dysgalactiae*. Furthermore, using this approach, we identified important AMR genes such as *Tet (A), Tet (38*), fosB-Saur, and blaZ. We showed that even 40 min of the MinION run was enough for bacterial identification and detecting the first AMR gene.

**Conclusion:**

We implemented an effective method (sensitivity of 100% and specificity of 92.3%) for host DNA removal and bacterial DNA enrichment (both gram-negative and positive) directly from bovine mastitis milk. To the best of our knowledge, this is the first culture- and amplification-independent study using nanopore-based metagenomic sequencing for real-time detection of the pathogen (within 5 hours) and the AMR profile (within 5–9 hours), in mastitis milk samples. These results provide a promising and potential future on-farm adaptable approach for better clinical management of mastitis.

## 1. Introduction

Mastitis is an inflammatory status of the mammary glands that can be caused by trauma and microbial invasion (Cheng and Han, 2020). Mastitis is often considered one of the main challenges in the dairy farm industry as it can impose substantial economic loss by decreasing milk production, reducing the chance of conception (Cheng and Han, 2020), negatively affecting animal welfare, and leading to financial losses (Ruegg, 2017). Based on data from the Norwegian Dairy Herd Recording System (NDHRS), clinical mastitis is the most frequently reported (ca. 34%) disease in Norwegian dairy cows (TINE, 2021). A total of 31,778 cases, corresponding to an incidence rate of 0.18 cases per cow per year, were reported in 2021 (TINE, 2021). The total corresponding loss was calculated to be ca. 103 Million NOK (TINE, 2021). Milk from treated cows must not be delivered to the dairy until it is free of drug residues for consumers’ sake (e.g., allergies, risk of developing AMR bacterial strains) and because antibiotics in milk can negatively impact the production of dairy products. Most bovine mastitis cases are caused by *Staphylococcus aureus*, non-aureus *staphylococci*, *Streptococcus* spp., *Escherichia coli*, and *Bacillus* spp. (Oliveira et al., 2013; Levison et al., 2016; Cheng and Han, 2020). *S. aureus* is the most frequent gram-positive pathogen causing several forms of clinical and sub-clinical mastitis in dairy cows (Abebe et al., 2016; Gomes et al., 2016; Cheng and Han, 2020). Previous studies also isolated *S. aureus* as the main invading pathogen in bovine mastitis in Norway (Østerås and Sølverød, 2009).

Antimicrobial resistance (AMR) has been recognized as one of the most threatening phenomena to public health, where bacteria no longer remain sensitive to antibiotics. The latest survey reported an estimated 1.27 million deaths directly and almost 5 million deaths associated with AMR in 2019 globally (Antimicrobial Resistance Collaborators, 2022). Another report estimated over 30,000 annual deaths in 2018 due to AMR only in Europe (OECD, 2018). The wide usage of antibiotics in mastitis is considered a potential risk to human health by increasing the risk of resistant strains occurring that may contaminate the food chain (Cheng and Han, 2020; Ma F. et al., 2021). This issue in *S. aureus* mastitis is even worse because of its unique characteristics, such as the immune escape (de Jong et al., 2019), subclinical pathogenicity (Campos et al., 2022), challenging eradication (Zaatout et al., 2020), and recurrency tendency (Miyazawa et al., 2022). Therefore, it is of great interest to rapidly diagnose relevant mastitis agent(s) in dairy cows, enabling the veterinarian to select the right antibiotics, and eventually helping farmers with a more reasonable livestock management strategy.

Conventional pathogen identification and antibiotic sensitivity assessment in mastitis are performed using culture-based methods. However, the traditional culture-dependent techniques are time-consuming and less sensitive to revealing the slow-growing bacteria. Hence such methods most likely underestimate invading agents and their AMR profiles. This could ultimately lead to more economic loss in dairy farms. Antimicrobial treatment is often initiated before culture results are present. Therefore, to minimize the risk of antibiotic resistance in mastitis, and to ensure better animal welfare, a rapid and accurate diagnosis of the primary pathogens and their antibiotic resistance profile is highly desirable.

Recent progress in high-throughput sequencing has led to an increasing interest in microbiota investigation of several biological ecosystems, including the bovine milk (McInnis et al., 2015; Addis et al., 2016; Catozzi et al., 2017; Catozzi et al., 2020). However, pathogen identification in mastitis milk using sequencing technologies is a challenging task because of the complexity of the milk sample and the high presence of host cells/floating host DNA. Such high background of host DNA most probably outnumbers the bacterial DNA reads and decreases the sensitivity of the method (McHugh et al., 2020). Several attempts have been made to minimize the host DNA in milk, such as sequencing at a high depth (Asnicar et al., 2017; Yap et al., 2020). However, such approaches could significantly increase the time needed and cost for pathogen identification, making it inappropriate for large numbers of samples (Thoendel et al., 2016). Therefore, a proper laboratory technique is required to minimize the presence of the host genome before direct sequencing from milk. There have been some recent investigations on clinical samples, including saliva, sputum, and joint fluid, to deplete host DNA through commercial kits or other chemical procedures (Thoendel et al., 2016; Marotz et al., 2018; Charalampous et al., 2019; Nelson et al., 2019). However, such approaches applied to milk samples are often limited to healthy/spiked samples and not actual mastitis milk samples (Rubiola et al., 2020; Yap et al., 2020; Siebert et al., 2021). Therefore, an effective and rapid method aiming to enrich the microbial DNA and deplete the host DNA, leading to accurate pathogen identification and AMR gene detection directly from the sample, is much needed.

This study aimed to deplete/minimize the host DNA and enrich the bacterial DNA in the bacterial-infected clinical mastitis milk sample. The second aim was to sequence the isolated DNA using real-time nanopore sequencing and identify the causative pathogens and AMR genes. Here, we have tested multiple strategies to enrich bacterial DNA and minimize the presence of bovine DNA in mastitis milk samples before direct (culture-independent) sequencing using the MinION platform. Using this method, we identified *S. aureus, E. coli*, and *Streptococcus dysgalactiae* as the primary causative pathogens and detected various AMR genes in mastitis milk samples within 5–9 h of sample collection.

## 2. Materials and methods

### 2.1. Raw milk samples

In this study, 22 milk samples, including 16 culture positive mastitis-infected and six culture negative non-mastitis from Norwegian red cows clinically diagnosed with *S. aureus* mastitis (10^3^–10^7^ CFU/ml), were collected and provided by TINE (Supplementary Table 1). In addition, another two samples (one flagged positive for *E. coli* and the other one flagged positive for both *E. coli* and *S. dysgalactiae*) were provided to test the most effective approach for microbial DNA isolation and host cell depletion. The initial screening and pathogen identification were performed in the TINE’s mastitis laboratory in Molde, Norway, using overnight culture, followed by PCR.

### 2.2. Bacterial DNA extraction procedures

Different strategies were used with various combinations of four commercial kits and techniques to isolate bacterial DNA from milk samples and deplete host cell DNA.

#### 2.2.1. Strategy A–testing different commercial kits without modification

We used DNeasy^®^ PowerFood^®^ Microbial Kit (Qiagen, Hilden, Germany) from here on PF_alone_, the Milk Bacterial DNA Isolation kit (Norgen Biotek Corp., Ontario, Canada) from here on NG_alone_, and the MolYsis™ Plus kit (Molzym, Bremen, Germany), from here on Mol Plus_alone_, according to manufacturers’ protocol. We followed some of the manufacturers’ recommendations for extra DNA yield for the PF Kit, including heating the cell lysate at 70°C for 10 min.

#### 2.2.2. Strategy B–testing a combination of milk filtering and different commercial kits

In this approach, and by taking advantage of size differences between microbial and mammalian cells, milk samples were first filtered using a sterile Acrodisc^®^ Syringe Filter with 5 μm Supor^®^ Membrane (Pall life science, Ann Arbor, USA). The filtered milk samples were further used for bacterial DNA isolation using PF, NG, and Mol Plus kits, according to the manufacturers’ recommendations (PF_filt_, NG_filt_, Mol Plus_filt_). The filtered milk was also plated on BHI agar plates to check if filtering affects *S. aureus* load.

#### 2.2.3. Strategy C–testing a combination of filtering, centrifugation, and different commercial kits

In this strategy, upon milk filtration, samples were centrifuged at a low speed (400 × *g* for 10 min at room temperature) to facilitate the sedimentation of the bovine cells. The supernatant was collected and used for bacterial DNA isolation using PF and NG kits according to the manufacturer’s recommendations (PF_filt–cent_, NG_filt–cent_). We also considered non-filtered but centrifuged milk samples for bacterial DNA isolation using the PF kit (PF_cent_).

#### 2.2.4. Strategy D–testing a combination of 10% Ox bile, nuclease, and different commercial kits

This approach tested mastitis milk samples using the PF, NG, Mol Plus, and MolYsis™ Complete5 kit (Molzym, Bremen, Germany) from here on Mol Com5. Experiments using the Mol Com5 kit were repeated twice.

For this purpose, aliquots (1 ml) of the same raw milk sample were first centrifuged at 4,500 × *g* for 20 min at 4°C. Then the supernatant was removed, and the pellet was washed twice in sterile Phosphate-buffered saline (PBS) and centrifuged at 13,000 × *g* for 1 min at room temperature, as described in Yap et al. (2020). The final pellet was resuspended in 1 ml sterile PBS and considered for bacterial DNA isolation as described below.

Four of the aliquots were considered for DNA isolation using Mol Plus and Mol Com5, with (Mol Plus_cent–nuc_, Mol Com5_cent–nuc_) and without (Mol Plus_cent_, Mol Com5_cent_) micrococcal nuclease (New England Biolab, Herts, UK) treatment. To reach an effective host DNA degradation, the micrococcal nuclease was added to the pellet obtained after mammalian cell lysis and the DNA degradation step in the MolYsis protocol. To do so, the pellet was mixed with 0.5 μl (10^3^ gel units) micrococcal nuclease in 100 μl reaction volume containing 10 μl nuclease reaction buffer, 0.5 μl Bovine serum albumin (BSA), filled up with nuclease-free water. Following incubation for 30 min at 37°C, enzyme deactivation was done by adding 100 μl of 20 mM ethylene glycol tetraacetic acid (EGTA). The suspension was washed twice in sterile PBS and centrifuged at 13,000 × *g* for 1 min at room temperature. The pellet was further used according to the rest of the MolYsis kits protocol.

Four other aliquots were incubated with 10% Ox bile for 10 min at room temperature. After incubation, the aliquots were centrifuged at 13,000 × *g* for 5 min, followed by two steps of washing with sterile PBS and centrifugation at 13,000 × *g* for 1 min. After that, DNA isolation using the four kits, in combination with micrococcal nuclease (PF_cent–ox–nuc_, NG_cent–ox–nuc_, Mol Plus_cent–ox–nuc_, Mol Com5_cent–ox–nuc_), was done as mentioned above.

### 2.3. DNA concentration and quality measurement

The DNA concentrations and purity were measured using a Qubit 2.0 Fluorometer (Thermo Fisher Scientific Inc., Waltham, MA, United States) and NanoDrop ND-1000 spectrophotometer (NanoDrop Technologies, Rockland, DE, United States) at wavelengths of 260 and 280 nm. Selected DNA isolates (seven mastitic and six negative samples for strategy A, seven mastitic samples for strategy C, two samples for strategy D, and two additional samples for testing the best strategy), despite their low concentration or purity (Supplementary Table 1), were submitted for PCR assay or library preparation for MinION sequencing.

### 2.4. Bacterial and host DNA identification using PCR

To confirm the host cell DNA depletion and bacterial DNA enrichment, two different sets of primers, including forward primer 5′-CTTGTATGAATGGCCGCACG-3′, reverse primer 5′-GATGTAGCGGGTCGTAGTGG-3′, targeting Bos taurus mitochondrion (NC_006853.1) and forward primer 5′-GGGTTGATACGCCAGAAACG-3′, reverse primer 5′-TGATGCTTCTTTGCCAAATGG-3′ targeting nuc gene (encodes thermonuclease in *S. aureus*), were designed using NCBI primer blast tool. Amplifications were carried out in a final volume of 20 μl PCR reaction containing 4 μl of HOT FIREPol^®^ Multiplex Mix Ready to Load (Solis Biodyne, Tartu, Estonia), 0.5 μl of each forward and reverse primers (10 μM), and volume corresponding to 1–10 ng/μl isolated DNA. Negative controls, consisting of PCR water instead of isolated DNA, were also considered.

The PCR reactions were performed using Veriti™ 96-Well Fast Thermal Cycler (Applied Biosystems, Foster City, CA, United States) with the following conditions: initial activation for 12 min at 95°C, 30 cycles of amplification at 95°C for 25 s, 60°C for 45 s, 72°C for 60 s, and a final elongation step at 72°C for 7 min. PCR products were run on 1% agarose electrophoresis, pre-mixed with SYBR^®^ safe DNA gel stain (Invitrogen, Carlsbad, CA, United States) for 45 min at 100 V. The PCR bands were visualized using the G: BOX Chemi XX6 gel documentation system (Syngene, Cambridge, United Kingdom).

### 2.5. MinION library preparation and sequencing

Before library preparation, Beckman Coulter™ Agencourt AMPure XP (Thermo Fisher Scientific) reagent was used to increase the DNA purity and concentration. Then the sequencing libraries were prepared using a rapid barcoding kit (SQK-RBK004) according to the manufacturer’s instructions.

Sequencing was performed using R9.4.1 flow cells (FLO-MIN106) mounted on a MinION device. The instrument was run for 96 h to acquire as much data as possible. Raw sequencing data were collected using ONT MinKNOW GUI software (version 5.0.0). The real-time base calling using FAST mode was also performed using ONT MinKNOW GUI software. Later, raw fast5 data were basecalled in the high accurate mode, demultiplexed, and trimmed for the barcodes/adapters using Guppy stand-alone software (version 6, Oxford Nanopore).

### 2.6. Bioinformatic analysis of MinION data

The Fastq files were converted to Fasta format, and fasta files were used for taxonomy identification and contaminant removal (host DNA) using Kraken2 in OmicsBox. Furthermore, the fasta files were Blast searched against RefSeq bacterial database. The antibiotic resistance genes were identified using ABRicate in default mode using NCBI AMRFinderPlus (Feldgarden et al., 2019; Seemann, 2020).

## 3. Results

### 3.1. Strategy A–testing different commercial kits without modification

**FIGURE 1:**
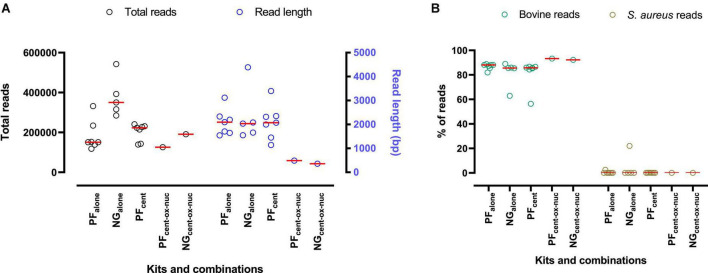
An overview of MinION sequencing results following DNA extraction from mastitis milk using PF/NG kits and different combinations. **(A)** Total reads (black circles) and read length (blue circles) following using different commercial kits/combinations. **(B)** Percentage of reads aligned with the bovine genome (green circles) and *Staphylococcus aureus* genome (olive circles). Each circle represents a sample, and the mean value is depicted with a red line.

The first set of experiments evaluated the efficiency of three commercial kits for bacterial DNA isolation from mastitis milk. Our data indicated that using PF_alone_ and NG_alone_, a high concentration of DNA was extracted (Table 1). MinION sequencing yielded a higher number of reads using NG_alone_ (Figure 1A). However, after taxonomic classification, we found that, on average, 86% (PF_alone_) and 81% (NG_alone_) of reads were aligned to the bovine genome (Figure 1B). On the other hand, only 0.3% (PF_alone_) and 4.5% (NG_alone_) of reads were found to be *S. aureus* reads (Figure 1B). The rest of the reads were either unclassified or belonged to other taxa. Our data also showed that Mol Plus_alone_ resulted in a very low concentration of isolated DNA, and no PCR band was detected for either bovine or *S. aureus* (Supplementary Figure 1 and Table 1). Furthermore, no *S. aureus* reads were identified in the negative samples, which is a good positive control. The negative samples were not considered for further analyses.

**TABLE 1:** An overview of a combination of kits and conditions to enrich *S. aureus* DNA and deplete bovine DNA directly from mastitis milk.

Strategy	Tested kit and modifications	*n*	Milk pre-filtered	Milk pre-centrifuged	Milk pretreated with Ox bile	Pellet treated with nuclease	DNA concentration (ng/μ l)	PCR product		MinION fastq data (GB)
								***S. aureus***	**Bovine**	
**A**	PF_alone_	7	–	–	–	–	98.7 ± 44.9	–	–	0.83 ± 0.47
	NG_alone_	5	–	–	–	–	61.4 ± 40.1	–	–	2 ± 1.64
	Mol plus_alone_	1	–	–	–	–	<0.2	ND	ND	–
**B**	PF_filt_	1	Yes	–	–	–	14.2	ND	**+ +++**	–
	NG_filt_	1	Yes	–	–	–	16	ND	**+ +++**	–
	Mol plus_filt_	1	Yes	–	–	–	<0.2	ND	ND	–
**C**	PF_cent_	7	–	Yes	–	–	87.2 ± 34.4	–	–	0.92 ± 0.44
	PF_filt–cent_	1	Yes	Yes	–	–	6.7	ND	**+ +++**	–
	NG_filt–cent_	1	Yes	Yes	–	–	1.6	ND	**+ +++**	–
**D**	PF_cent–ox–nuc_	1	–	Yes*	Yes	Yes	100	**+**	**+++ +**	0.19
	NG_cent–ox–nuc_	1	–	Yes*	Yes	Yes	99.8	**+ +**	**+++ +**	0.49
	Mol plus_cent_	1	–	Yes*	–	–	<0.2	ND	ND	0.005
	Mol plus_cent–nuc_	1	–	Yes*	–	Yes	<0.2	**+ ++**	**++**	0.005
	Mol plus_cent–ox–nuc_	1	–	Yes*	Yes	Yes	<0.2	**+ +++**	ND	0.14
	Mol Com5_cent_	2	–	Yes*	–	–	97.2 ± 3.8	**+ ++**	**+++**	0.08 ± 0.05
	Mol Com5_cent–nuc_	2	–	Yes*	–	Yes	7.7 ± 8.1	**+ +++**	**+**	0.50 ± 0.26
	Mol Com5_cent–ox–nuc_	2	–	Yes*	Yes	Yes	6.0 ± 7.3	**+ +++**	**+**	0.56 ± 0.26

The strategies are highlighted by different background colors. The abbreviation describing each combination and condition is given in the kits and combination column. Data presented as mean ± SD. The – symbol indicates that the step or experiment was not performed. ND indicates no PCR band was detected and the + sign shows the intensity of the PCR band (product). The Yes sign shows the low-speed centrifugation (400 × *g* for 10 min at room temperature) and the Yes* sign shows centrifugation at 4,500 × *g* for 20 min at 4°C followed by two washing steps in PBS. The number of samples is given by *n*.

### 3.2. Strategy B–testing a combination of milk filtering and different commercial kits

This experiment aimed to reduce the bovine cells in the milk samples before DNA isolation. Data presented in Table 1 indicates that filtering remarkably affected the concentration of isolated DNA for both PF_filt_ (14.2 ng/μl) and NG_filt_ (16 ng/μl), compared to strategy A (PF_alone_ = 98.7 ng/μl, NG_alone_ = 61.4 ng/μl). The lower bacterial load following filtering was further confirmed by CFU counting (Supplementary Table 2). We did not observe any difference in concentration of isolated DNA following using Mol Plus_filt_ compared to Mol Plus_alone_ (both yielded low DNA concentration, <0.2 ng/μl). Like strategy A, we did not observe any bovine or *S. aureus* PCR band following using Mol Plus_filt_. However, after using PF_filt_ and NG_filt_, a strong bovine PCR band was observed, while the *S. aureus* band was again missing (Table 1 and Supplementary Figure 1).

### 3.3. Strategy C–testing a combination of filtering, centrifugation, and different commercial kits

Before DNA isolation, we employed a low-speed centrifugation step to improve host cell removal from filtrate mastitis milk. As can be seen from Table 1, DNA concentration even further decreased following a combination of filtering and low-speed centrifugation in PF_filt–cent_ (6.7 ng/μl) and NG_filt–cent_ (1.6 ng/μl), compared to PF_filt_ (14.2 ng/μl) and NG_filt_ (16 ng/μl) and PF_alone_ (98.7 ng/μl) and NG_alone_ (61.4 ng/μl). However, we observed no PCR band for *S. aureus*, while a bovine PCR band was identified using PF_filt–cent_ and NG_filt–cent_. Low-speed centrifugation of mastitis milk without filtration (PF_cent_) also resulted in a high percentage of bovine reads (81.5%) without remarkably affecting the *S. aureus* reads enrichment (0.08%) (Figure 1B). We also tested different low-speed centrifugation conditions. The data showed that increasing the time of low-speed centrifugation reduced the bacterial load in the samples (Supplementary Table 2).

### 3.4. Strategy D–testing a combination of 10% Ox bile, nuclease, and different commercial kits

To effectively facilitate bacterial release from bovine cells and deplete host cell and bovine DNA degradation, we implemented a washing step in combination with adding 10% Ox bile and micrococcal nuclease. PCR assay following DNA isolation using PF_cent–ox–nuc_ and NG_cent–ox–nuc_ indicated a clear improvement in *S. aureus* band intensity, while bovine PCR product was also identified (Table 1 and Supplementary Figure 1).

**FIGURE 2:**
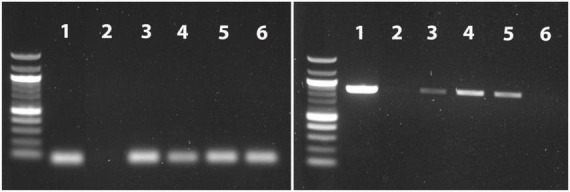
An overview of PCR results following DNA extraction directly from mastitis milk. PCR bands for the *nuc* gene in *S. aureus* **(left)** and *Bos taurus* mitochondrion **(right)** were visualized in their expected size of 65 and 879 bp, respectively. The 100 bp ladder was also used.1: Mol Com5_cent_, 2: Mol Plus_cent_, 3: Mol Com5_cent–nuc_, 4: Mol Plus_cent–nuc_, 5: Mol Com5_cent–ox–nuc_, 6: Mol Plus_cent–ox–nuc_.

We also tested different combinations of two different kits from MolYsis™ and micrococcal nuclease with or without 10% Ox bile. None of the bovine or *S. aureus* DNA was detected when we used Mol Plus_cent_ (Figure 2, band 2). We identified both *S. aureus* and bovine DNA *via* PCR assay almost with similar band intensity following using Mol com5_cent_ (Figure 2, bands 1). However, the PCR bands for bovine were much weaker when we used Mol com 5_cent–nuc_ (Figure 2, band 3) and Mol com 5_cent–ox–nuc_ (Figure 2, band 5). Interestingly, the PCR band for bovine DNA almost disappeared for Mol Plus_cent–ox–nuc_ (Figure 2, band 6). As appeared in Figure 2, the intensity for the PCR band corresponding to *S. aureus* DNA was identical for Mol com 5_cent–nuc_, Mol com 5_cent–ox–nuc_, and Mol Plus_cent–ox–nuc_.

**FIGURE 3:**
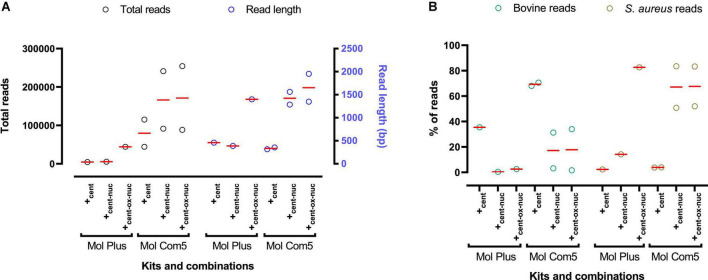
An overview of MinION sequencing results following DNA extraction from mastitis milk using MolYsis kits and combinations. **(A)** Total reads (black) and read length (blue) following using different commercial kits/combinations. **(B)** Percentage of reads aligned with the bovine genome (green) and *S. aureus* genome (olive). Each circle represents a sample, and the mean value is depicted with a red line.

Furthermore, results showed that the Mol Plus kit in all combinations yielded a very low DNA concentration (similar to Mol Plus_alone_ in strategy A and Mol Plus_filt_ in strategy B) (Table 1). Surprisingly, the sequencing data indicated a low number of unexpected short-length reads (<500 bp) following using Mol Plus_cent_, Mol Plus_cent–nuc_ (Figure 3A), PF_cent–ox–nuc_, and NG_cent–ox–nuc_ (Figure 1A). However, after using a combination of Ox bile and nuclease (Mol Plus_cent–ox–nuc_), we observed a long read within the expected range for MinION (Figure 3A) and a strong PCR product band (Figure 2, band 6) only for *S. aureus*, indicating successful depletion of the host cell and DNA degradation. Kraken2 analyses further confirmed these results. As can be seen from Figure 3B, and following using Mol Plus_cent–ox–nuc_ combination, we observed a high percentage of *S. aureus* reads (82%) and a low percentage of bovine reads contamination (2.5%). Although we observed a weak band for *S. aureus* following using the PF_cent–ox–nuc_ and NG_cent–ox–nuc_ (Supplementary Figure 1), the MinION data indicated a high degree of host DNA contamination (92–93%) for both kits/combinations (Figure 1B).

Comparing Mol com5 and Mol Plus kits, the Mol com5 kit gave higher DNA concentration and more data from MinION sequencing in all tested conditions (Table 1 and Figure 3A). Similar to Mol Plus_cent_ and Mol Plus_cent–nuc_, we noticed a short read length (<500 bp) for Mol com5_cent_ (Figure 3A). However, in contrast to Mol Plus_cent–nuc_, adding nuclease resulted in long MinION reads (Figure 3A). Similar long reads to Mol Plus_cent–ox–nuc_ were also observed when we applied Ox bile (Mol com 5_cent–ox–nuc_) (Figure 3A).

As can be seen from Figure 3B, combining Mol Com5 with nuclease and Ox bile effectively reduced host DNA reads and increased *S. aureus* reads. On average and following using both Mol Com 5_cent–nuc_ and Mol Com 5_cent–ox–nuc_, we were able to reduce bovine reads from 68% (for Mol Com 5_cent_) to 17% and increase *S. aureus* reads from 3.5 (for Mol Com 5_cent_) to 66.5%. These results show that both Mol Com5_cent–ox–nuc_ and Mol com5_cent–nuc_ are effective approaches with a sensitivity of 100% and a specificity of 92.3% for bacterial DNA enrichment and microbial/AMR genes identification (see Supplementary Table 1 for details).

### 3.5. AMR gene detection following effective host cell depletion and bacterial DNA enrichment

**FIGURE 4:**
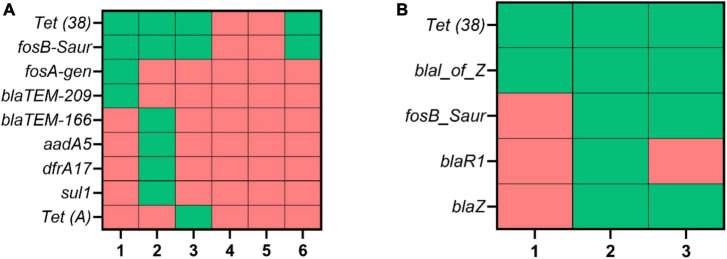
An overview of identified AMR genes, following MinION DNA sequencing. Two mastitis milk samples **(A,B)** were subjected to bacterial DNA isolation using different kits and conditions (culture-independent) combinations. The green and red cells show the presence and absence of AMR genes, respectively. 1: Mol Com5_cent_, 2: Mol Com5_cent–nuc_, 3: Mol Com5_cent–ox–nuc_, 4: Mol Plus_cent_, 5: Mol Plus_cent–nuc_, 6: Mol Plus_cent–ox–nuc_.

One of the main goals of this study was to use sequenced DNA for AMR gene detection in mastitis milk. As shown in Figure 4, the highest number of AMR genes was detected using Mol Com5_cent–nuc_ for both replicates. We were not able to detect any AMR genes for Mol Plus_cent_ and Mol Plus_cent–nuc_, where we had a low concentration of DNA. However, such a low concentration of DNA was observed for Mol Plus_cent–ox–nuc_, yet we could detect two AMR genes, including *tet (38*) and *fosB-Saur*. Both these genes were detected using all combinations for the Mol com5 kit as well.

### 3.6. MinION sequencing for pathogen identification and AMR gene detection in mastitis milk

**FIGURE 5:**
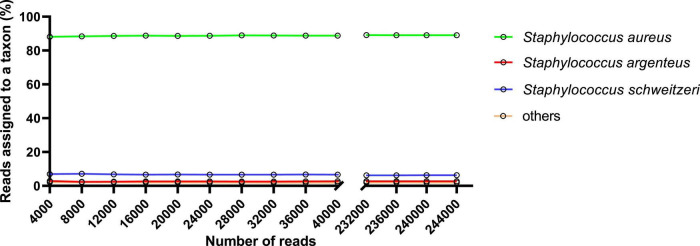
A representative of BLAST analyses for taxon assigned reads. Data was obtained following DNA sequencing for DNA isolated using the Mol Com5_cent–nuc_ approach (one of the replicates).

Our data further showed that using BLAST analyses, the first 4,000 reads were enough for *S. aureus* identification (close to 90% similarity). The percentage of similarity remained consistent throughout the sequencing (Figure 5).

**FIGURE 6:**
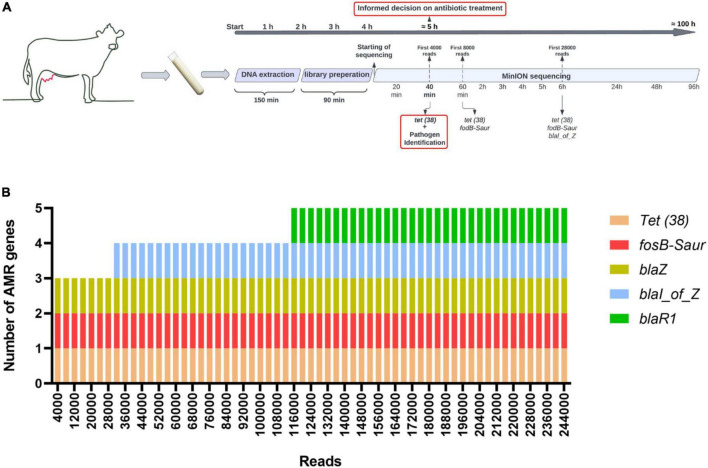
Study workflow and comparison of the amount of data needed for AMR detection following two different base calling modes. Data was obtained following DNA sequencing for the Mol Com5_cent–nuc_ approach (one of the replicates). **(A)** Timing of experiment and time/reads required for AMR gene detection following FAST base calling and using real-time data analyses. **(B)** Number of AMR genes detected per amount of data, following high accuracy base calling for the same sample.

As shown in Figure 6A, the whole process, from sample delivery to starting the sequencing, took only 4 h. Furthermore, we took advantage of real-time MinION data analyses using our in-house developed pipeline. The results showed that the first 4,000 and 8,000 reads that were base called using the FAST mode were obtained just in 40 and 60 min and were enough for *tet (38*) and *fosB-Saur* identification, respectively. We noticed that 6 h of sequencing was enough to identify the *blal_of_Z* gene (Figure 6A). When we used stand-alone guppy to base call the reads using high accuracy mode, the first 4,000 reads were enough for *tet (38*), *fosB-Saur*, and *blaZ* genes. Other AMR genes, including *blal_of_Z* and *blaR1*, were identified when 32,000 and 116,000 reads were base called, respectively (Figure 6B).

### 3.7. Identifying other bovine mastitis pathogens using Mol Com5_cent–ox–nuc_ combination

We tested the most promising method (Mol Com5_cent–ox–nuc_) using two other milk samples from mastitis animals (Supplementary Figure 2). Following culturing the samples in the microbiology lab, one was flagged positive for *E. coli*, and the other was flagged positive for both *Streptococcus dysgalactiae* and *E. coli*. Following DNA isolation using the Mol Com5_cent–ox–nuc_ approach, the PCR experiment showed strong bands for *E. coli* (first sample) and only *S*. *dysgalactiae* (second sample). Sequencing results showed 2 and 3% host contamination for the first and second samples, respectively. In addition, for the first sample and following BLAST search against the RefProk database, 30% of taxon were reported to be *E. coli* reads, which agrees with PCR and visual culture characteristic results. Worth mentioning that several reads of the taxon for the first sample were reported to be *Shigella* spp. reads. We blasted reads flagged as *Shigella* against the *E. coli* genome database and identified hits with over 80% similarity and vice versa. For the second sample, BLAST analyses indicated that most reads are from *S*. *dysgalactiae*, and no reads were identified as *E. coli.* The second predominant taxon (11%) in the second sample was *Enterococcus faecalis*. This agrees with PCR results and is in contrast with visual culture characteristics.

## 4. Discussion

In the current study, we tested different kits and combinations to effectively reduce the host DNA and enrich bacterial DNA from milk samples from cows flagged positive for mastitis.

### 4.1. Food-specific bacterial DNA isolation kits yielded a lower number of *S. aureus* reads

We have tested two of the well-known bacterial isolation kits (PF and NG) designed to isolate bacterial DNA from food matrices. The sequencing results indicated neither of these kits could effectively deplete the host DNA and/or enrich the bacterial DNA (Figure 1). Using PF_alone_ and NG_alone_, on average, over 90% of the reads were classified as bovine reads in negative samples. The high percentage of host DNA in this study for mastitis samples (over 80% using PF_alone_, NG_alone_, PF_cent_, and close to 70% using Mol Com5_cent_) is similar to the previously investigated milk microbiome using other kits such as MolYsis Basic5, NEBNext, and PowerSoil Pro kits (Rubiola et al., 2020; Yap et al., 2020). To our knowledge, neither PF nor NG kit contained reagents designed specifically for host cell depletion or host DNA degradation. On the other hand, it has been observed that *S. aureus* is characterized by an intracellular localization in mammary gland epithelial cells during mastitis (Kerro Dego et al., 2002). Therefore, an effective host cell lysis is crucial for rapidly detecting the pathogen and AMR genes in a complex environment like milk, where a high degree of host cell/DNA exists. Most previous research that used PF (Ganda et al., 2016; Lima et al., 2018; Taponen et al., 2019; Al-Harbi et al., 2021; Ma T. et al., 2021; Schwenker et al., 2022) or NG kit (Quigley et al., 2012; Cheema et al., 2021; Lyons et al., 2021) considered 16S metagenomic sequencing for bacterial identification following DNA extraction; therefore, no host DNA contamination was reported. While amplicon sequencing is a sensitive method for taxonomy clarification, the relevance of this approach for rapid diagnostic and AMR gene detection in mastitis is questionable. However, applying PF and NG kits for metagenomic sequencing of mastitis milk samples did not give the desired results in this study.

### 4.2. Low-speed centrifugation and pre-filtration remarkably reduced the concentration of isolated DNA but did not deplete host DNA

Milk filtration, with or without low-speed centrifugation, decreased the concentration of isolated DNA and did not enrich bacterial DNA fraction. These results might be explained by the fact that some of the bacteria might have adhered to the fat globules and milk proteins, therefore, cannot pass through the filtration or be discarded after centrifugation. Filtration and centrifugation did not contribute to host DNA removal. Similar results were previously reported in a study where the authors observed a high percentage of host-aligned reads in DNA extracted from the human saliva filtrate, despite applying a 5 μm filter and differential centrifugation (Marotz et al., 2018). These results are likely related to the presence of extracellular DNA in the sample environment, which was previously highlighted as a challenge in metagenomic sequencing for microbial DNA in human samples (Shi et al., 2022). Data from several studies suggest that some of the toxins produced by invading bacteria in mastitis could cause host cell damage (Mehrzad et al., 2005; Oviedo-Boyso et al., 2007). For instance, previous evidence shows that enterotoxin M and H from *S. aureus* cause necrosis and apoptosis of bovine mammary epithelial cells to a high extent (Liu et al., 2014; Zhao et al., 2020). Therefore, host cell necrosis/apoptosis likely results in host DNA release into the milk environment, which cannot be minimized using filtration or low-speed centrifugation.

### 4.3. MolYsis kits yielded low DNA concentration and relatively shorter MinION reads

We could not detect the bovine or *S. aureus* PCR band using the Mol Plus_alone_ (Figure 2). This was further confirmed by the low quantity of isolated DNA (<0.2 ng/μl) in all tested combinations for this kit (Table 1). Low DNA concentration obtained using the MolYsis™ Plus kit agrees with earlier findings (Villumsen et al., 2010; Anson et al., 2018) and those used other MolYsis™ variants such as MolYsis™ Basic5 (Rubiola et al., 2020) and MolYsis™ Complete5 kits (Yap et al., 2020).

It is also worth mentioning that the MolYsis™ Plus kit is designed to be used for bacterial DNA isolation from whole blood, which may not be appropriate for other specimen types, such as milk. We also observed a relatively shorter MinION read length (<500 bp) when using Mol Plus_cent_, Mol Plus_cent–nuc_, and Mol Com5_cent_ (Figure 3A). The average expected read length of bacterial DNA for the MinION rapid barcoding kit in our previous research was reported to be close to 3,500 bp (Taxt et al., 2020; Khezri et al., 2021). A short read length combined with a low DNA concentration for the MolYsis™ Plus kit indicates that it degrades both the host and the bacterial DNA to a greater extent. Shorter reads could cause an extra challenge in taxonomy classification and AMR gene detection. Interestingly, when we used the two MolYsis™ kits in combination with micrococcus nuclease, a similar short read length was observed only for MolYsis™ Plus and not for MolYsis™ Complete5 (Figure 3A). The reason for this is not apparent. However, this difference may be due to the difference in composition of the two kits, which is proprietary information. Therefore, these results need to be interpreted with caution, and further research should be undertaken using a higher number of samples.

### 4.4. Pre-treatment of milk with Ox bile, enriched bacterial DNA, resulted in longer reads

The Ox bile improved the read length when using MolYsis™ kits (Figure 3A). It has been suggested that Ox bile has a lysing effect on the human host cell and facilitates the pathogen release from the human blood cells (Zhou and Pollard, 2010; Zhou and Pollard, 2012). As stated before, *S. aureus* is mainly localized in epithelial cells of mammary glands. Therefore, it seems possible that Ox bile facilitated the *S. aureus* release from the bovine epithelial cells (floating in the mastitis milk samples) in this study. Previous published data, indicated that optimal host cell lysis and degradation of background DNA following using reagents supplied with commercial kits are challenging, therefore most of the previous research used an additional step of host cell lysis using other types of buffers, such as saponin (Hasan et al., 2016; Bruggeling et al., 2021; Siebert et al., 2021). Although it has been shown that the majority of pathogens are inherently tolerant against Ox bile (Begley et al., 2005), one needs to keep in mind that current promising results might not be applicable for the enrichment of other microorganisms such as mycoplasma which doesn’t have a cell wall to protect genetic material.

### 4.5. MolYsis kits, in combination with Ox bile and nuclease, effectively depleted the host DNA and enriched the bacterial DNA

The present study showed that the lysis of host cells, followed by the degradation of extracellular DNA, can potentially enrich the bacterial DNA and reduce the background bovine DNA. Here, pre-incubating the milk samples with 10% Ox bile, combined with micrococcal nuclease and MolYsis™ Plus or MolYsis™ complete5, both effectively depleted host DNA and increased the number, as well as the length of *S. aureus*, reads (Figures 3A, B). However, since DNA concentration using Mol Plus_cent–ox–nuc_ was much lower than Mol Com5_cent–ox–nuc_, which could compromise library preparation, we preferred Mol com5_cent–ox–nuc_ over Mol Plus_cent–ox–nuc_ in this study. Adding 10% Ox bile slightly improved the results when using Mol com5_cent–ox–nuc_ compared with Mol com5_cent–nuc_. This might be because of lysing effect of Ox bile, which is in line with previous research where authors showed that combining saponin (as a lysing reagent) and DNase is more effective than DNase alone (Hasan et al., 2016). Here, the DNA concentration and A260/230 ratios following using both Ox bile and nuclease were low (Supplementary Table 1). The current low DNA concentration and purity results agree with previously published data where authors applied six different protocols to isolate bacterial DNA from bovine milk diagnosed with diseased udder (Schwenker et al., 2022). This might be explained by the complexity of milk samples, especially in clinical mastitis cases.

Previous research reported that the MolYsis™ complete5 kit effectively reduced the host DNA contamination down to 60% in bovine milk from healthy cows (Yap et al., 2020). It is worth mentioning that the authors also reported a high degree of variation between samples. Here, we effectively deplete the host DNA down to almost 80% in mastitis milk using the MolYsis™ complete5 kit in combination with nuclease or Ox bile + nuclease. However, the amount of isolated DNA from this approach was lower than DNA concentrations using other described methods, which makes sequencing library preparation challenging (Shi et al., 2022). Furthermore, when we used a combination of Ox bile, Mol Com5, and nuclease, we observed a discrepancy among two replicates regarding the percentage of aligned reads with Bovine and *S. aureus* (Figure 3B). This inconsistency was previously also reported for MolYsis™ complete5 kit (Yap et al., 2020). It may be due to the decrease in quality of the original specimen (due to frequent freezing and thawing) and/or unequal sensitivities of DNA to the lysing conditions, as discussed here (Cressier and Bissonnette, 2011; Hasan et al., 2016; Shi et al., 2022). One way to minimize this variation is to work with fresh milk samples and avoid frequent freezing and thawing, which might compromise the efficacy of DNA isolation (Shi et al., 2022).

The combination of MolYsis™ complete5 kit, Ox bile + nuclease was shown to be an effective approach to isolate not only gram positive but also gram-negative bacterial DNA. Although we observed some degree of misclassification between *E. coli* and *Shigella* spp. for the first tested sample, the distinction between *Shigella* spp. and *E. coli* is a well-known challenge in microbiology as they share many biochemical, phenotypic, and genetic properties. The genus *Shigella* comprises several clusters interspersed in the *E. coli* phylogeny (Taxt et al., 2020).

### 4.6. Ox bile and micrococcal nuclease are not compatible with PF and/or NG kits

Although combining the Ox bile and micrococcal nuclease with both MolYsis™ kits resulted in long MinION reads and effective host DNA depletion, the combination was not compatible with PF and/or NG kits. Both PF_cent–ox–nuc_ and NG_cent–ox–nuc_ did not effectively deplete the host DNA, and PCR results indicated a weak *S. aureus* band and a strong bovine PCR band (Supplementary Figure 1) and resulted in short MinION reads (<500 bp). According to the results presented here, Ox bile and micrococcal nuclease could lead to inconsistent results when combined with different commercial kits. The reason for this is not clear. However, it could potentially be due to the incompatibility of the kit’s component (which is proprietary information and not available in the public domain) with Ox bile and micrococcal nuclease.

### 4.7. Effective host cell depletion and DNA degradation improved the prediction of AMR genes

A prerequisite for successfully detecting AMR genes using sequencing technology is the high-quality data that cannot be achieved in the presence of high host DNA reads. In this study, the reduction of host DNA was linked with a greater number of AMR determinants (except for Mol Plus_cent–ox–nuc_) (Figure 4). Among the methods tested, the combination of MolYsis Complete5 kit and micrococcal nuclease resulted in the highest number of AMR genes detected in milk samples. We identified two AMR genes, including *tet (38*) and *fosB-sur*, giving resistance to tetracycline and fosfomycin, using Mol Plus_cent–ox–nuc_. It is worth mentioning that both *tet (38*) and *fosB-sur* were identified using all other combinations except Mol Plus_cent_ and Mol Plus_cent–nuc_, where we observed a low DNA concentration (Table 1). Our results showed some cases of discrepancy between phenotypic AMR and genotypic data, where we detected different variants of the *bla* gene. However, this is not surprising as the disagreement between genotypic and phenotypic data previously reported in bacteria (Zankari et al., 2012; Marotta et al., 2020; Avershina et al., 2021) and could be explained by the fact that a phenotypic resistance might be related to multiple different genes. A reasonable approach to tackle this issue could be developing machine learning algorithms that predict resistance in clinical isolates. We recently have developed such algorithms for WHO high-priority pathogens, including *E. coli* and *K. pneumoniae* which can be easily adapted for mastitis samples (Avershina et al., 2021).

### 4.8. MinION sequencing is a promising approach for rapid diagnosis of pathogen and antibiotic resistance profiles as well as better clinical management of mastitis

A growing body of literature emphasized the applicability of MinION sequencing and its advantages for the infectious disease diagnostics (Petersen et al., 2019; Mongan et al., 2020; Taxt et al., 2020; Avershina et al., 2021; Ciuffreda et al., 2021). However, the use of MinION sequencing in food microbiology and veterinary medicine fields so far has been very limited to a few studies (Catozzi et al., 2020; Azinheiro et al., 2021; Shinozuka et al., 2021). Identifying causative pathogens in mastitis-infected animals is a challenging task, and previous research has documented that some of the causative pathogens are not detectable using conventional diagnostic methods (Taponen et al., 2009; Kuehn et al., 2013; Addis et al., 2016). Here we were able to significantly shorten the time required for pathogen identification (both gram-positive and negative) and AMR gene detection (<9 h from sample delivery). Furthermore, our data showed how MinION sequencing could provide extra diagnostic confidence when there is a disagreement between colony morphology and PCR results. Although similar promising results were previously reported for blood (Taxt et al., 2020) and urine (Zhang et al., 2022) specimens, this is the first study that reported how to handle mastitis milk samples for effective and rapid pathogen and AMR detection.

## 5. Conclusion

Current research found both Mol Com5_cent–ox–nuc_ and Mol Com5_cent–nuc_ as effective methods having very high sensitivity and specificity and minimal differences for bacterial identification and AMR gene detection in clinical mastitis milk samples. Adding 10% Ox bile requires an extra hour for library preparation and may reduce the concentration of extracted DNA. Therefore, considering minimal differences and high workload for Mol Com5_cent–ox–nuc_, it seems reasonable to prioritize the Mol Com5_cent–nuc_ approach. We have shown how nanopore sequencing coupled with an effective method for bacterial DNA enrichment and real-time bioinformatics analysis could result in better clinical management of single and potentially mixed-pathogen mastitis. Moreover, using this method, we can identify the primary causative pathogens (within 5 h) and detect various AMR genes (within 5–9 h). To the best of our knowledge, no previous study has implemented WGS for pathogen identification and AMR detection following direct DNA extraction from milk (culture and amplification independent approach) in bovine mastitis. This method provides several advantages, including data from clinical mastitis milk samples (not artificially contaminated or spiked), sensitivity for both gram-positive and gram-negative pathogens, the minimal infrastructure needed for making sequencing libraries, portability of MinION, and rapid data analysis, making the current approach appropriate even for the future on-farm approach for rapid diagnosis of mastitis and AMR testing. A limitation of this study was the small sample size and using the exact same sample(s) to test different strategies. Future studies would focus on a larger sample size spread across different pathogens (e.g., *Streptococcus agalactiae*, *Streptococcus uberis*, and *Enterococcus* spp.) and AMR profiles (e.g., *tet*, *aphA*, and other β-lactamases). This would provide the reliability of the described approach for potential use for real-time diagnosis, thereby facilitating efficient antimicrobial stewardship and preventing the spread of AMR. Our study shows that direct sequencing of clinical mastitis samples without culture and amplification could provide a rapid diagnostic method for better clinical management of mastitis.

## Data availability statement

The datasets presented in this study can be found in online repositories. The names of the repository/repositories and accession number(s) can be found below: https://www.ebi.ac.uk/ena, PRJEB53532.

## Author contributions

HN facilitated the sampling from infected cows and contributed to the clinical mastitis discussions. AA, AK, and RA wrote the manuscript. AA performed the experimental work in discussions with AK and RA. AK and AA analyzed the data in discussions with RA. All authors designed the experiments and read and edited the manuscript.

## Supplementary material

Supplementary Figure 1 PCR bands for *nuc* gene in *S. aureus* and *Bos taurus* mitochondrion following DNA extraction using the different combinations of kits and conditions.

Supplementary Figure 2 PCR primer information and results for *E. coli* and *S. dysgalactiae* following testing the Mol Com5_cent–ox–nuc_ approach for two test samples.

Supplementary Table 1 An overview of samples and their respective DNA concentration and purity parameters following different DNA isolation conditions.

Supplementary Table 2 Effect of centrifugation and filtration on bacterial load of mastitis milk samples.

## References

[B1] Abebe R. Hatiya H. Abera M. Megersa B. Asmare K. (2016). Bovine mastitis: Prevalence, risk factors and isolation of *Staphylococcus aureus* in dairy herds at Hawassa milk shed, South Ethiopia. *BMC Vet. Res.* 12:270. 10.1186/s12917-016-0905-3 27912754 PMC5135792

[B2] Addis M. Tanca A. Uzzau S. Oikonomou G. Bicalho R. Moroni P. (2016). The bovine milk microbiota: Insights and perspectives from-omics studies. *Mol. Biosyst.* 12 2359–2372. 10.1039/c6mb00217j 27216801

[B3] Al-Harbi H. Ranjbar S. Moore R. Alawneh J. (2021). Bacteria isolated from milk of dairy cows with and without clinical mastitis in different regions of Australia and their AMR profiles. *Front. Vet. Sci.* 8:743725. 10.3389/fvets.2021.743725. 34805335 PMC8600363

[B4] Anson L. Chau K. Sanderson N. Hoosdally S. Bradley P. Iqbal Z. (2018). DNA extraction from primary liquid blood cultures for bloodstream infection diagnosis using whole genome sequencing. *J. Med. Microbiol.* 67 347–357. 10.1099/jmm.0.000664 29458686 PMC5882078

[B5] Antimicrobial Resistance Collaborators (2022). Global burden of bacterial antimicrobial resistance in 2019: A systematic analysis. *Lancet* 399 629–655. 35065702 10.1016/S0140-6736(21)02724-0 PMC8841637

[B6] Asnicar F. Manara S. Zolfo M. Truong D. Scholz M. Armanini F. (2017). Studying vertical microbiome transmission from mothers to infants by strain-level metagenomic profiling. *mSystems* 2 e164–e116. 10.1128/mSystems.00164-16 28144631 PMC5264247

[B7] Avershina E. Sharma P. Taxt A. Singh H. Frye S. Paul K. (2021). AMR-Diag: Neural network based genotype-to-phenotype prediction of resistance towards β-lactams in *Escherichia coli* and *Klebsiella pneumoniae*. *Comput. Struct. Biotechnol. J.* 19 1896–1906. 10.1016/j.csbj.2021.03.027 33897984 PMC8060595

[B8] Azinheiro S. Roumani F. Carvalho J. Prado M. Garrido-Maestu A. (2021). Suitability of the MinION long read sequencer for semi-targeted detection of foodborne pathogens. *Anal. Chim. Acta* 1184:339051. 10.1016/j.aca.2021.339051 34625270

[B9] Begley M. Gahan C. Hill C. (2005). The interaction between bacteria and bile. *FEMS Microbiol. Rev.* 29 625–651. 10.1016/j.femsre.2004.09.003 16102595

[B10] Bruggeling C. Garza D. Achouiti S. Mes W. Dutilh B. Boleij A. (2021). Optimized bacterial DNA isolation method for microbiome analysis of human tissues. *Microbiologyopen* 10:e1191. 10.1002/mbo3.1191 34180607 PMC8208965

[B11] Campos B. Pickering A. Rocha L. Aguilar A. Fabres-Klein M. de Oliveira Mendes T. (2022). Diversity and pathogenesis of Staphylococcus aureus from bovine mastitis: Current understanding and future perspectives. *BMC Vet. Res.* 18:115. 10.1186/s12917-022-03197-5 35331225 PMC8944054

[B12] Catozzi C. Ceciliani F. Lecchi C. Talenti A. Vecchio D. De Carlo E. (2020). Short communication: Milk microbiota profiling on water buffalo with full-length 16S rRNA using nanopore sequencing. *J. Dairy Sci.* 103 2693–2700. 10.3168/jds.2019-17359 31980229

[B13] Catozzi C. Sanchez Bonastre A. Francino O. Lecchi C. De Carlo E. Vecchio D. (2017). The microbiota of water buffalo milk during mastitis. *PLoS One* 12:e0184710. 10.1371/journal.pone.0184710 28926595 PMC5604978

[B14] Charalampous T. Kay G. Richardson H. Aydin A. Baldan R. Jeanes C. (2019). Nanopore metagenomics enables rapid clinical diagnosis of bacterial lower respiratory infection. *Nat. Biotechnol.* 37 783–792. 10.1038/s41587-019-0156-5 31235920

[B15] Cheema A. Stinson L. Lai C. Geddes D. Payne M. S. D. N. A. (2021). Extraction method influences human milk bacterial profiles. *J. Appl. Microbiol.* 130 142–156. 10.1111/jam.14780 32654260

[B16] Cheng W. Han S. (2020). Bovine mastitis: Risk factors, therapeutic strategies, and alternative treatments–A review. *Asian Australas. J. Anim. Sci.* 33 1699–1713. 10.5713/ajas.20.0156 32777908 PMC7649072

[B17] Ciuffreda L. Rodríguez-Pérez H. Flores C. (2021). Nanopore sequencing and its application to the study of microbial communities. *Comput. Struct. Biotechnol. J.* 19 1497–1511. 10.1016/j.csbj.2021.02.020 33815688 PMC7985215

[B18] Cressier B. Bissonnette N. (2011). Assessment of an extraction protocol to detect the major mastitis-causing pathogens in bovine milk. *J. Dairy Sci.* 94 2171–2184. 10.3168/jds.2010-3669 21524507

[B19] de Jong N. W. M. van Kessel K. van Strijp J. (2019). Immune evasion by *Staphylococcus aureus*. *Microbiol. Spectr.* 7 1–27. 10.1128/microbiolspec.gpp3-0061-2019 PMC11590434 30927347

[B20] Feldgarden M. Brover V. Haft D. Prasad A. Slotta D. Tolstoy I. (2019). Validating the AMRFinder tool and resistance gene database by using antimicrobial resistance genotype-phenotype correlations in a collection of isolates. *Antimicrob. Agents Chemother.* 63 e483–e419. 10.1128/AAC.00483-19 PMC6811410 31427293

[B21] Ganda E. Bisinotto R. Lima S. Kronauer K. Decter D. Oikonomou G. (2016). Longitudinal metagenomic profiling of bovine milk to assess the impact of intramammary treatment using a third-generation cephalosporin. *Sci. Rep.* 6:37565. 10.1038/srep37565 27874095 PMC5118806

[B22] Gomes F. Saavedra M. Henriques M. (2016). Bovine mastitis disease/pathogenicity: Evidence of the potential role of microbial biofilms. *Pathog. Dis.* 74:ftw006. 10.1093/femspd/ftw006 26772653

[B23] Hasan M. Rawat A. Tang P. Jithesh P. Thomas E. Tan R. (2016). Depletion of human DNA in spiked clinical specimens for improvement of sensitivity of pathogen detection by next-generation sequencing. *J. Clin. Microbiol.* 54 919–927. 10.1128/JCM.03050-15 26763966 PMC4809942

[B24] Kerro Dego O. van Dijk J. Nederbragt H. (2002). Factors involved in the early pathogenesis of bovine *Staphylococcus aureus* mastitis with emphasis on bacterial adhesion and invasion. A review. *Vet. Q.* 24 181–198. 10.1080/01652176.2002.9695135 12540135

[B25] Khezri A. Avershina E. Ahmad R. (2021). Hybrid assembly provides improved resolution of plasmids, antimicrobial resistance genes, and virulence factors in *Escherichia coli* and *Klebsiella pneumoniae* clinical isolates. *Microorganisms* 9:2560. 10.3390/microorganisms9122560 34946161 PMC8704702

[B26] Kuehn J. Gorden P. Munro D. Rong R. Dong Q. Plummer P. (2013). Bacterial community profiling of milk samples as a means to understand culture-negative bovine clinical mastitis. *PLoS One* 8:e61959. 10.1371/journal.pone.0061959 23634219 PMC3636265

[B27] Levison L. Miller-Cushon E. Tucker A. Bergeron R. Leslie K. Barkema H. (2016). Incidence rate of pathogen-specific clinical mastitis on conventional and organic Canadian dairy farms. *J. Dairy Sci.* 99 1341–1350. 10.3168/jds.2015-9809 26686728

[B28] Lima S. Bicalho M. Bicalho R. (2018). Evaluation of milk sample fractions for characterization of milk microbiota from healthy and clinical mastitis cows. *PLoS One* 13:e0193671. 10.1371/journal.pone.0193671 29561873 PMC5862444

[B29] Liu Y. Chen W. Ali T. Alkasir R. Yin J. Liu G. (2014). Staphylococcal enterotoxin H induced apoptosis of bovine mammary epithelial cells in vitro. *Toxins* 6 3552–3567. 10.3390/toxins6123552 25533519 PMC4280547

[B30] Lyons K. Fouhy F. Ca O. Ryan C. Dempsey E. Ross R. (2021). Effect of storage, temperature, and extraction kit on the phylogenetic composition detected in the human milk microbiota. *Microbiologyopen* 10:e1127. 10.1002/mbo3.1127 33373099 PMC7841076

[B31] Ma F. Xu S. Tang Z. Li Z. Zhang L. (2021). Use of antimicrobials in food animals and impact of transmission of antimicrobial resistance on humans. *Biosaf. Health* 3 32–38.

[B32] Ma T. Shen L. Wen Q. Lv R. Hou Q. Kwok L. (2021). PacBio sequencing revealed variation in the microbiota diversity, species richness and composition between milk collected from healthy and mastitis cows. *Microbiology* 167 1–10. 10.1099/mic.0.000968 34292863

[B33] Marotta F. Di Marcantonio L. Janowicz A. Pedonese F. Di Donato G. Ardelean A. (2020). Genotyping and antibiotic resistance traits in *Campylobacter jejuni* and coli from pigs and wild boars in Italy. *Front. Cell. Infect. Microbiol.* 10:592512. 10.3389/fcimb.2020.592512. 33178635 PMC7593542

[B34] Marotz C. Sanders J. Zuniga C. Zaramela L. Knight R. Zengler K. (2018). Improving saliva shotgun metagenomics by chemical host DNA depletion. *Microbiome.* 6:42. 10.1186/s40168-018-0426-3 29482639 PMC5827986

[B35] McHugh A. Feehily C. Fenelon M. Gleeson D. Hill C. Cotter P. (2020). Tracking the dairy microbiota from farm bulk tank to skimmed milk powder. *mSystems* 5 e226–e220. 10.1128/mSystems.00226-20 32265313 PMC7141888

[B36] McInnis E. Kalanetra K. Mills D. Maga E. (2015). Analysis of raw goat milk microbiota: Impact of stage of lactation and lysozyme on microbial diversity. *Food Microbiol.* 46 121–131. 10.1016/j.fm.2014.07.021 25475275

[B37] Mehrzad J. Desrosiers C. Lauzon K. Robitaille G. Zhao X. Lacasse P. (2005). Proteases involved in mammary tissue damage during endotoxin-induced mastitis in dairy cows. *J. Dairy Sci.* 88 211–222. 10.3168/jds.S0022-0302(05)72679-5 15591384

[B38] Miyazawa R. Shimoda S. Matsuda K. Tobe R. Ando T. Yoneyama H. (2022). Characterization of *Staphylococcus aureus* isolates from bovine mastitis and bulk tank milk: First isolation of methicillin-susceptible *Staphylococcus aureus* in Japan. *Microorganisms* 10:2117. 10.3390/microorganisms10112117 36363708 PMC9696108

[B39] Mongan A. Tuda J. Runtuwene L. (2020). Portable sequencer in the fight against infectious disease. *J. Hum. Genet.* 65 35–40. 10.1038/s10038-019-0675-4 31582773 PMC6892364

[B40] Nelson M. Pope C. Marsh R. Wolter D. Weiss E. Hager K. (2019). Human and extracellular DNA depletion for metagenomic analysis of complex clinical infection samples yields optimized viable microbiome profiles. *Cell Rep.* 26 2227.e–2240.e. 10.1016/j.celrep.2019.01.091 30784601 PMC6435281

[B41] OECD (2018). *Antimicrobial resistance, tackling the burden in the European union.* Paris: OECD Publishing.

[B42] Oliveira L. Hulland C. Ruegg P. (2013). Characterization of clinical mastitis occurring in cows on 50 large dairy herds in Wisconsin. *J. Dairy Sci.* 96 7538–7549. 10.3168/jds.2012-6078 24119795

[B43] Østerås O. Sølverød L. (2009). Norwegian mastitis control programme. *Ir. Vet. J.* 62:S26. 10.1186/2046-0481-62-S4-S26 PMC3339347 22081877

[B44] Oviedo-Boyso J. Valdez-Alarcón J. Cajero-Juárez M. Ochoa-Zarzosa A. López-Meza J. Bravo-Patiño A. (2007). Innate immune response of bovine mammary gland to pathogenic bacteria responsible for mastitis. *J. Infect.* 54 399–409. 16882453 10.1016/j.jinf.2006.06.010

[B45] Petersen L. Martin I. Moschetti W. Kershaw C. Tsongalis G. (2019). Third-generation sequencing in the clinical laboratory: Exploring the advantages and challenges of nanopore sequencing. *J. Clin. Microbiol.* 58 e01315–e01319. 10.1128/JCM.01315-19 31619531 PMC6935936

[B46] Quigley L. O’Sullivan O. Beresford T. Paul Ross R. Fitzgerald G. Cotter P. D. A. (2012). Comparison of methods used to extract bacterial DNA from raw milk and raw milk cheese. *J. Appl. Microbiol.* 113 96–105. 22452460 10.1111/j.1365-2672.2012.05294.x

[B47] Rubiola S. Chiesa F. Dalmasso A. Di Ciccio P. Civera T. (2020). Detection of antimicrobial resistance genes in the milk production environment: Impact of host DNA and sequencing depth. *Front. Microbiol.* 11:1983. 10.3389/fmicb.2020.01983 32983010 PMC7479305

[B48] Ruegg P. L. A. (2017). 100-Year review: Mastitis detection, management, and prevention. *J. Dairy Sci.* 100 10381–10397. 10.3168/jds.2017-13023 29153171

[B49] Schwenker J. Friedrichsen M. Waschina S. Bang C. Franke A. Mayer R. (2022). Bovine milk microbiota: Evaluation of different DNA extraction protocols for challenging samples. *MicrobiologyOpen* 11:e1275. 10.1002/mbo3.1275 35478279 PMC9059235

[B50] Seemann T. (2020). *Abricate.* Available online at: https://github.com/tseemann/abricate (accessed December 15, 2022).

[B51] Shi Y. Wang G. Lau H. Yu J. (2022). Metagenomic sequencing for microbial DNA in human samples: Emerging technological advances. *Int. J. Mol. Sci.* 23:2181. 10.3390/ijms23042181 PMC8877284 35216302

[B52] Shinozuka Y. Kawai K. Kurumisawa T. Shimizu Y. Imanishi T. Ohno A. (2021). Examination of the microbiota of normal cow milk using MinION(TM) nanopore sequencing. *J. Vet. Med. Sci.* 83 1620–1627. 10.1292/jvms.21-0353 34526421 PMC8636880

[B53] Siebert A. Hofmann K. Staib L. Doll E. Scherer S. Wenning M. (2021). Amplicon-sequencing of raw milk microbiota: Impact of DNA extraction and library-PCR. *Appl. Microbiol. Biotechnol.* 105 4761–4773. 10.1007/s00253-021-11353-4 34059942 PMC8195793

[B54] Taponen S. McGuinness D. Hiitiö H. Simojoki H. Zadoks R. Pyörälä S. (2019). Bovine milk microbiome: A more complex issue than expected. *Vet. Res.* 50:44. 10.1186/s13567-019-0662-y 31171032 PMC6555717

[B55] Taponen S. Salmikivi L. Simojoki H. Koskinen M. Pyörälä S. (2009). Real-time polymerase chain reaction-based identification of bacteria in milk samples from bovine clinical mastitis with no growth in conventional culturing. *J. Dairy Sci.* 92 2610–2617. 10.3168/jds.2008-1729 19447993

[B56] Taxt A. Avershina E. Frye S. Naseer U. Ahmad R. (2020). Rapid identification of pathogens, antibiotic resistance genes and plasmids in blood cultures by nanopore sequencing. *Sci. Rep.* 10:7622. 10.1038/s41598-020-64616-x PMC7203151 32376847

[B57] Thoendel M. Jeraldo P. Greenwood-Quaintance K. Yao J. Chia N. Hanssen A. (2016). Comparison of microbial DNA enrichment tools for metagenomic whole genome sequencing. *J. Microbiol. Methods* 127 141–145. 27237775 10.1016/j.mimet.2016.05.022 PMC5752108

[B58] TINE (2021). *The annual report for cows and goat based on the health card records.* Available online at: https://medlem.tine.no/fag-og-forskning/statistikksamling-for-ku-og-geitekontrollen-2021 (accessed December 15, 2022).

[B59] Villumsen S. Pedersen R. Krogfelt K. Jensen J. (2010). Expanding the diagnostic use of PCR in leptospirosis: Improved method for DNA extraction from blood cultures. *PLoS One* 5:e12095. 10.1371/journal.pone.0012095 20711446 PMC2920309

[B60] Yap M. Feehily C. Walsh C. Fenelon M. Murphy E. McAuliffe F. (2020). Evaluation of methods for the reduction of contaminating host reads when performing shotgun metagenomic sequencing of the milk microbiome. *Sci. Rep.* 10:21665. 10.1038/s41598-020-78773-6 33303873 PMC7728742

[B61] Zaatout N. Ayachi A. Kecha M. (2020). *Staphylococcus aureus* persistence properties associated with bovine mastitis and alternative therapeutic modalities. *J. Appl. Microbiol.* 129 1102–1119. 10.1111/jam.14706 32416020

[B62] Zankari E. Hasman H. Kaas R. Seyfarth A. Agersø Y. Lund O. (2012). Genotyping using whole-genome sequencing is a realistic alternative to surveillance based on phenotypic antimicrobial susceptibility testing. *J. Antimicrob. Chemother.* 68 771–777. 10.1093/jac/dks496 23233485

[B63] Zhang L. Huang W. Zhang S. Li Q. Wang Y. Chen T. (2022). Rapid detection of bacterial pathogens and antimicrobial resistance genes in clinical urine samples with urinary tract infection by metagenomic nanopore sequencing. *Front. Microbiol.* 13:858777. 10.3389/fmicb.2022.858777 35655992 PMC9152355

[B64] Zhao Y. Tang J. Yang D. Tang C. Chen J. (2020). Staphylococcal enterotoxin M induced inflammation and impairment of bovine mammary epithelial cells. *J. Dairy Sci.* 103 8350–8359. 32622596 10.3168/jds.2019-17444

[B65] Zhou L. Pollard A. J. A. (2010). Fast and highly sensitive blood culture PCR method for clinical detection of *Salmonella enterica* serovar Typhi. *Ann. Clin. Microbiol. Antimicrob.* 9:14. 10.1186/1476-0711-9-14 20403166 PMC2873252

[B66] Zhou L. Pollard A. J. A. (2012). Novel method of selective removal of human DNA improves PCR sensitivity for detection of *Salmonella Typhi* in blood samples. *BMC Infect. Dis.* 12:164. 10.1186/1471-2334-12-164 22839649 PMC3482578

