# Supplementary material for: A culture-, amplification-independent, and rapid method for identification of pathogens and antibiotic resistance profile in bovine mastitis milk
Source: Front Microbiol. 2023 Jan 6;13:1104701. doi: 10.3389/fmicb.2022.1104701 (PMC9852903; doi:10.3389/fmicb.2022.1104701)
Supplement: Supplementary Figure 1 — PCR bands for nuc gene in S. aureus and Bos taurus mitochondrion following DNA extraction using the different combinations of kits and conditions. [file Data_Sheet_1.zip › Supplementary Figure 1.pdf]

Supplementary Figure 1. PCR bands for *nuc* gene in *S. aureus* and *Bos taurus* mitochondrion following DNA extraction using different combination of kits and conditions.

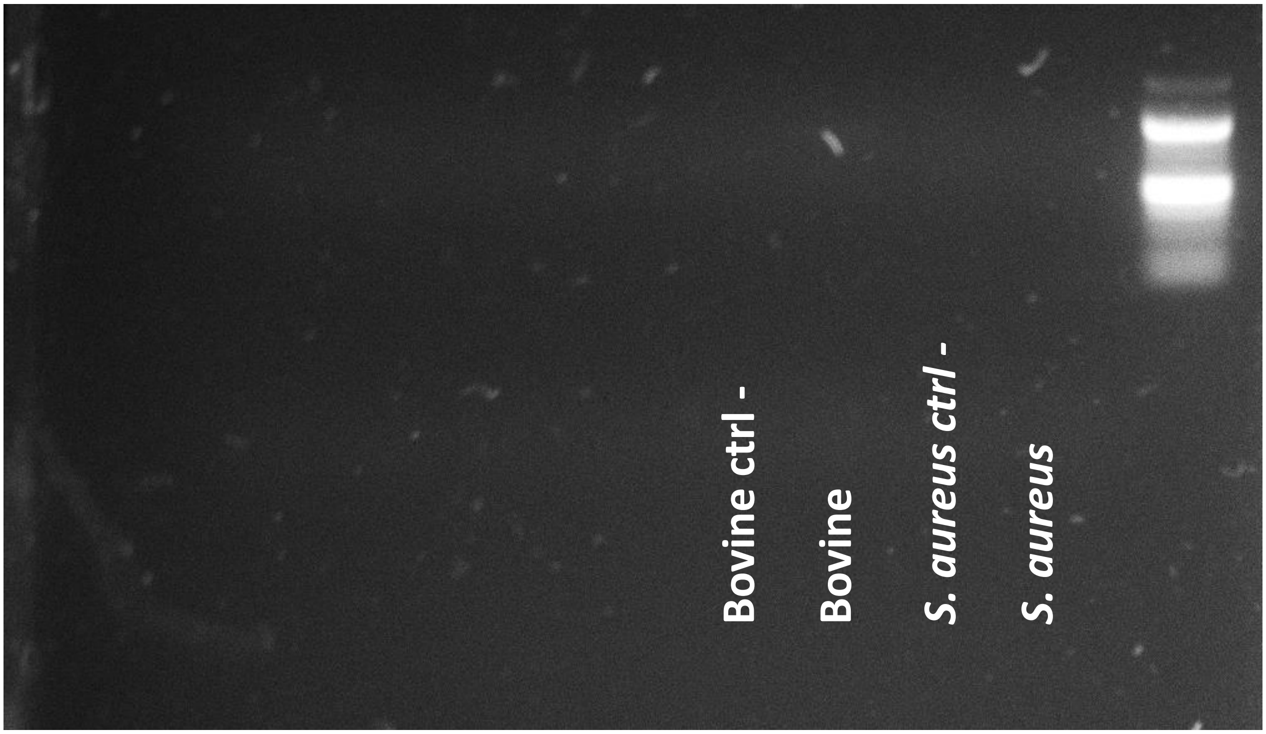

Supplementary Figure 1-A. PCR bands for *nuc* gene in *S. aureus* and *Bos taurus* mitochondrion following DNA extraction using Mol plus $_{alone}$ (strategy A). For ctrl negative samples, PCR water was considered instead of isolated DNA in PCR reaction.

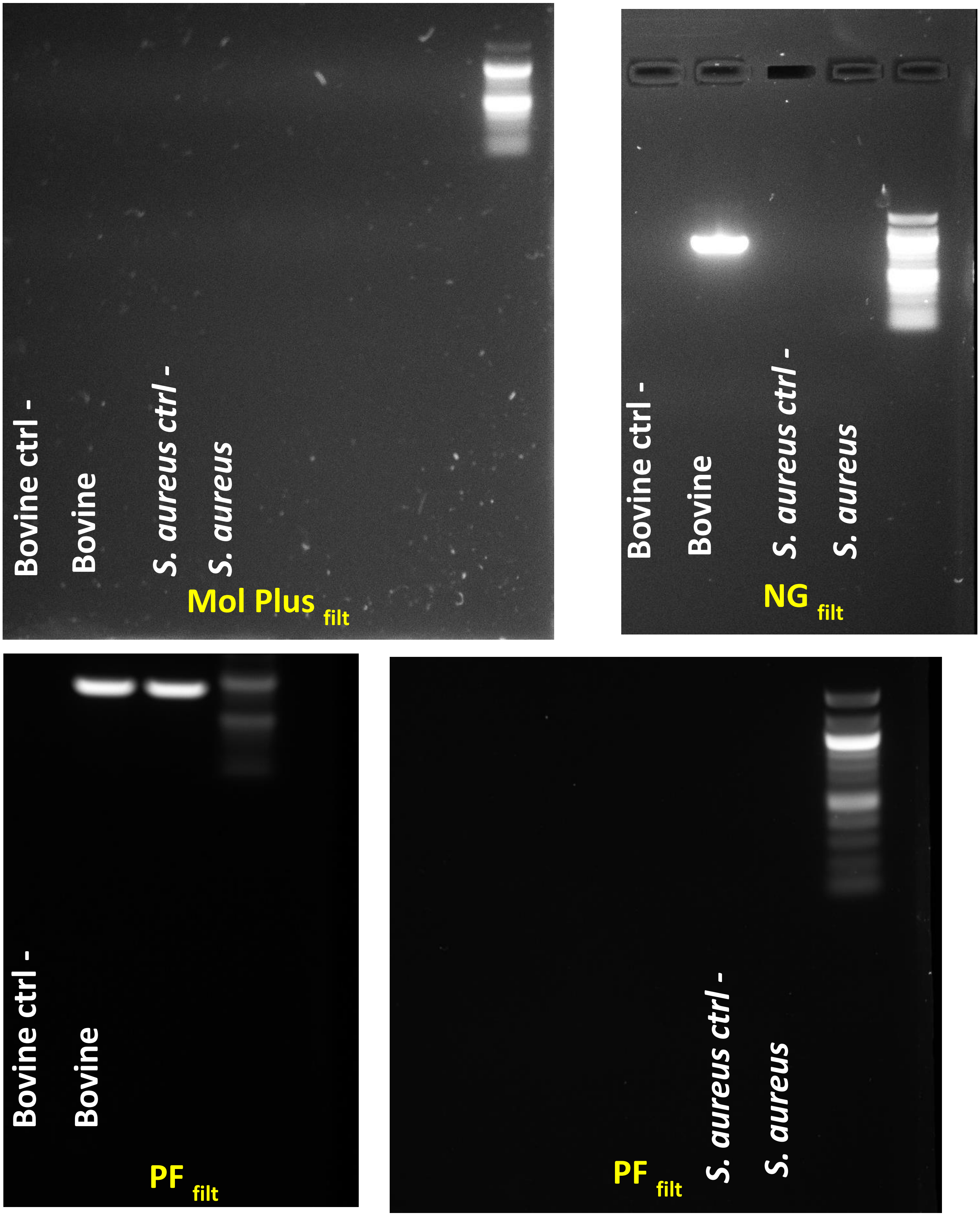

Supplementary Figure 1-B. PCR bands for nuc gene in S. aureus and Bos taurus mitochondrion following DNA extraction using PF filt, NG filt, Mol Plus filt (strategy B). For ctrl negative samples, PCR water was considered instead of isolated DNA in PCR reaction.

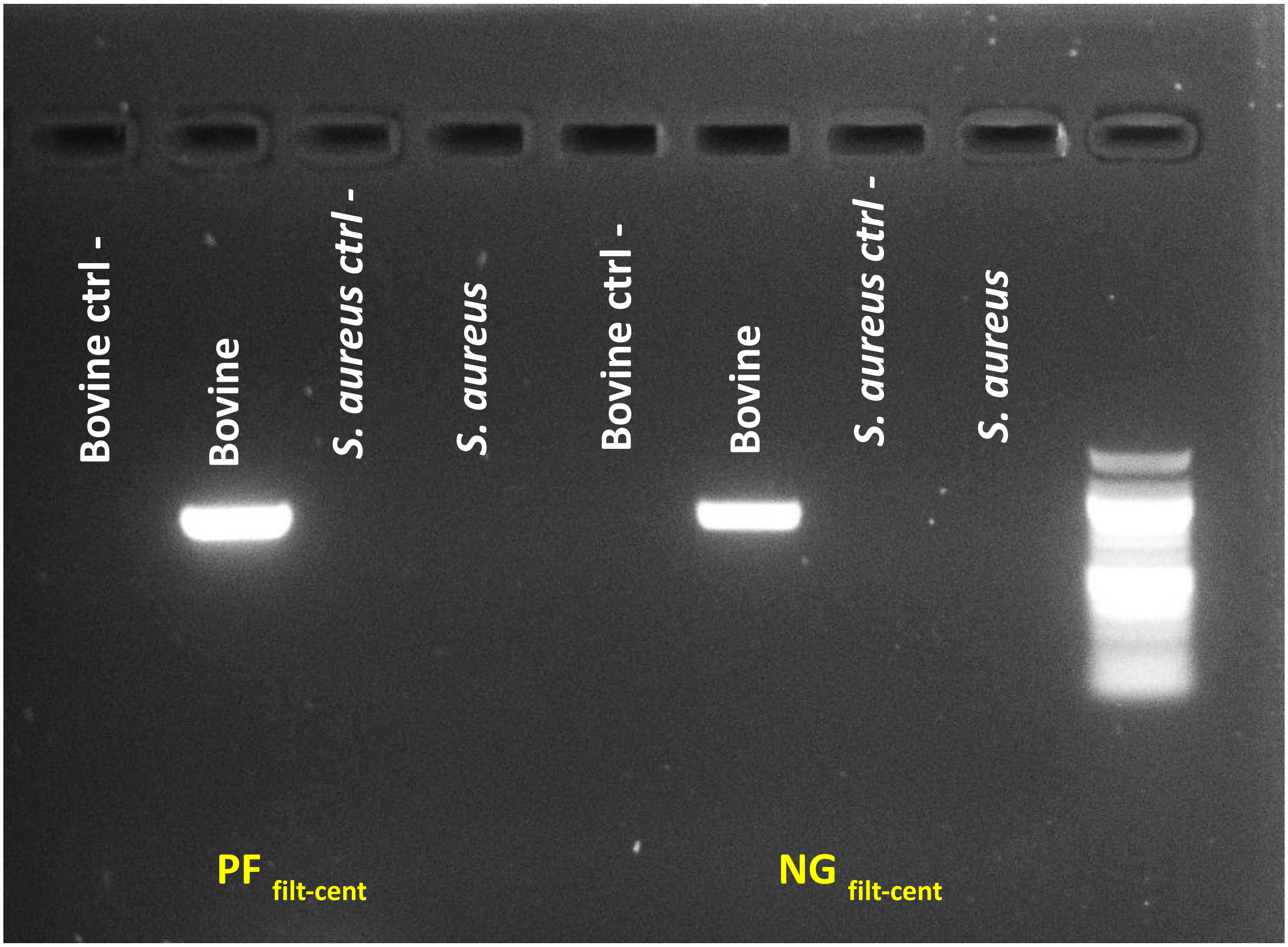

Supplementary Figure 1-C. PCR bands for *nuc* gene in *S. aureus* and *Bos taurus* mitochondrion following DNA extraction using PF $_{\text{filt-cent}}$, NG $_{\text{filt-cent}}$ (strategy C). For ctrl negative samples, PCR water was considered instead of isolated DNA in PCR reaction.

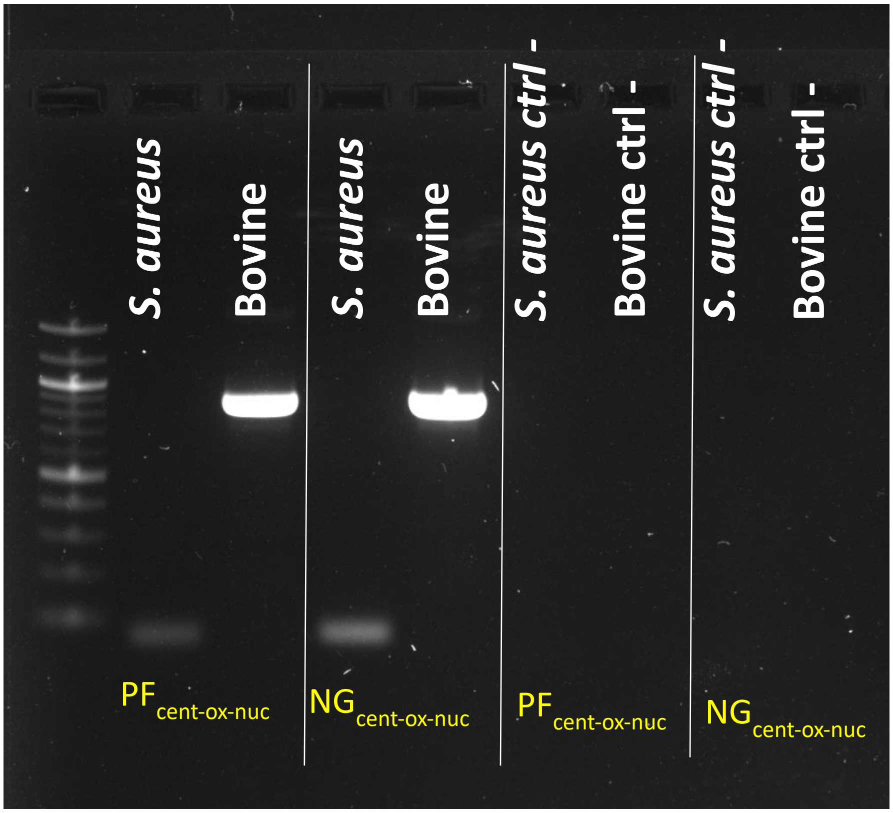

Supplementary Figure 1-D. PCR bands for *nuc* gene in *S. aureus* and *Bos taurus* mitochondrion following DNA extraction using PF $_{\text{cent-ox-nuc}}$, NG $_{\text{cent-ox-nuc}}$ (strategy D). For ctrl negative samples, PCR water was considered instead of isolated DNA in PCR reaction.
