# Supplementary material for: A culture-, amplification-independent, and rapid method for identification of pathogens and antibiotic resistance profile in bovine mastitis milk
Source: Front Microbiol. 2023 Jan 6;13:1104701. doi: 10.3389/fmicb.2022.1104701 (PMC9852903; doi:10.3389/fmicb.2022.1104701)
Supplement: Supplementary Figure 1 — PCR bands for nuc gene in S. aureus and Bos taurus mitochondrion following DNA extraction using the different combinations of kits and conditions. [file Data_Sheet_1.zip › Supplementary Figure 2.pdf]

Supplementary Figure 2. PCR primer information and results for two tested samples.

Primer was designed using NCBI primer blast tool against 16S rRNA gene in *Streptococcus dysgalactiae* (gene bank accession nr OP067819.1). Expected PCR product length: 149 bp

Forward GCAAGTAGAACGCTGAGGACT

Reverse ATGAAAAACATGGGTCTTCCATTGT

Primers were designed against universal stress protein (uspA) gene in *E. Coli* and taken from (Chen and Griffiths, 1998). Expected PCR product length: 884 bp

Forward CCGATACGCTGCCAATCAGT

Reverse ACGCAGACCGTAGGCCAGAT

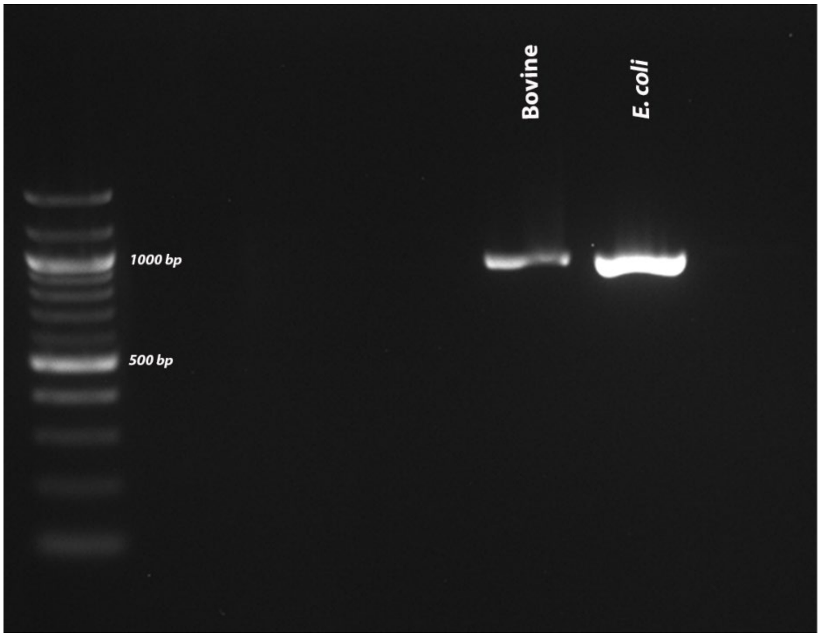

Sample 1

Sample 1 (concentration of isolated DNA: 0.252 ng/ul with Qubit, 8.2 ng/ul with Nanodrop) was a mastitis milk in which *E. coli* colonies were isolated and confirmed using PCR.

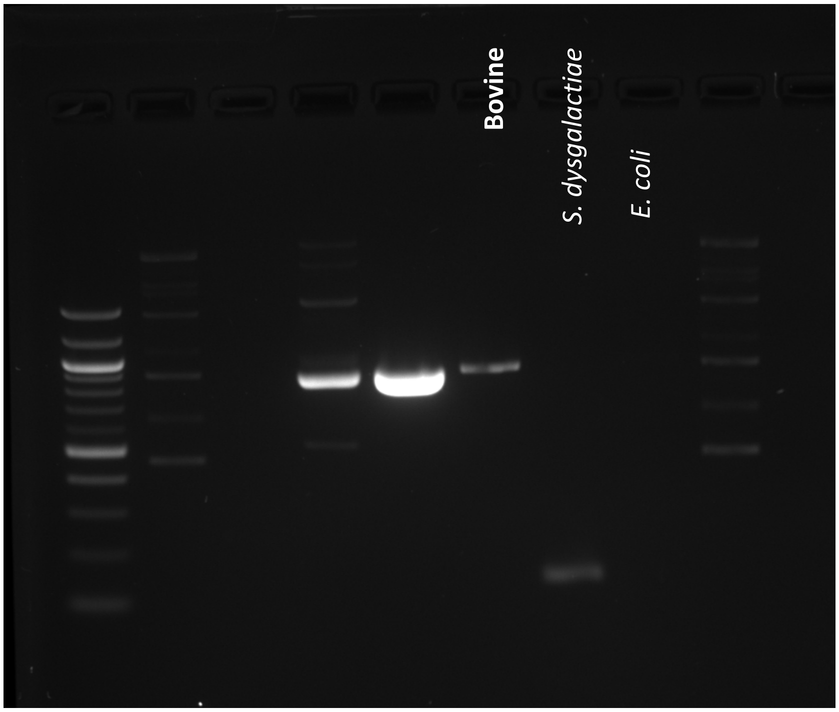

Sample 2

For sample 2 (concentration of isolated DNA: < 0.2 ng/ul with Qubit, 5.8 ng/ul with Nanodrop), both *E. coli* and *S. dysgalactiae* were identified following culture, but no PCR bands were observed for *E. coli*. In sample 2, for this study, lanes corresponding to Bovine, *S. dysgalactiae,* and *E. coli* were the 6th, 7th, and 8th lanes, respectively. Lanes 2 – 5 in the figure belonged to a different experiment.

References:

Chen, J., and M.W. Griffiths. 1998. PCR differentiation of Escherichia coli from other gram-negative bacteria using primers derived from the nucleotide sequences flanking the gene encoding the universal stress protein. *Lett Appl Microbiol*. 27:369-371.
